# Alcohol Intake and Alcohol–SNP Interactions Associated with Prostate Cancer Aggressiveness

**DOI:** 10.3390/jcm10030553

**Published:** 2021-02-02

**Authors:** Hui-Yi Lin, Xinnan Wang, Tung-Sung Tseng, Yu-Hsiang Kao, Zhide Fang, Patricia E. Molina, Chia-Ho Cheng, Anders E. Berglund, Rosalind A. Eeles, Kenneth R. Muir, Nora Pashayan, Christopher A. Haiman, Hermann Brenner, Jong Y. Park, The PRACTICAL Consortium

**Affiliations:** 1 Biostatistics Program, School of Public Health, Louisiana State University Health Sciences Center, New Orleans, LA 70112, USA; x.wang1@medpace.com (X.W.); zfang@lsuhsc.edu (Z.F.); 2 Behavioral and Community Health Sciences Program, School of Public Health, Louisiana State University Health Sciences Center, New Orleans, LA 70112, USA; ttseng@lsuhsc.edu (T.-S.T.); ykao1@lsuhsc.edu (Y.-H.K.); 3 Department of Physiology, School of Medicine, Louisiana State University Health Sciences Center, New Orleans, LA 70112, USA; PMolin@lsuhsc.edu; 4 Comprehensive Alcohol Research Center, School of Medicine, Louisiana State University Health Sciences Center, New Orleans, LA 70112, USA; 5 Biostatistics and Bioinformatics Shared Resource, H. Lee Moffitt Cancer Center and Research Institute, Tampa, FL 33612, USA; Chia-Ho.Cheng@moffitt.org; 6 Department of Biostatistics and Bioinformatics, H. Lee Moffitt Cancer Center and Research Institute, Tampa, FL 33612, USA; Anders.Berglund@moffitt.org; 7 The Institute of Cancer Research, and The Royal Marsden NHS Foundation Trust, London SM2 5NG, UK; Ros.Eeles@icr.ac.uk; 8 Division of Population Health, Health Services Research, and Primary Care, University of Manchester, Oxford Road, Manchester M13 9PT, UK; kenneth.muir@manchester.ac.uk; 9 Department of Applied Health Research, University College London, London WC1E 7HB, UK; n.pashayan@ucl.ac.uk; 10 Center for Genetic Epidemiology, Department of Preventive Medicine, Keck School of Medicine, University of Southern California, Los Angeles, CA 90015, USA; Haiman@usc.edu; 11 Norris Comprehensive Cancer Center, Los Angeles, CA 90015, USA; 12 Division of Clinical Epidemiology and Aging Research, German Cancer Research Center (DKFZ), D-69120 Heidelberg, Germany; h.brenner@dkfz.de; 13 Division of Preventive Oncology, German Cancer Research Center (DKFZ) and National Center for Tumor Diseases (NCT), D-69120 Heidelberg, Germany; 14 German Cancer Consortium (DKTK), German Cancer Research Center (DKFZ), D-69120 Heidelberg, Germany; 15 Department of Cancer Epidemiology, H. Lee Moffitt Cancer Center and Research Institute, Tampa, FL 33612, USA; Jong.Park@moffitt.org

**Keywords:** alcohol intake, SNP interaction, prostate cancer

## Abstract

Excessive alcohol intake is a well-known modifiable risk factor for many cancers. It is still unclear whether genetic variants or single nucleotide polymorphisms (SNPs) can modify alcohol intake’s impact on prostate cancer (PCa) aggressiveness. The objective is to test the alcohol–SNP interactions of the 7501 SNPs in the four pathways (angiogenesis, mitochondria, miRNA, and androgen metabolism-related pathways) associated with PCa aggressiveness. We evaluated the impacts of three excessive alcohol intake behaviors in 3306 PCa patients with European ancestry from the PCa Consortium. We tested the alcohol–SNP interactions using logistic models with the discovery-validation study design. All three excessive alcohol intake behaviors were not significantly associated with PCa aggressiveness. However, the interactions of excessive alcohol intake and three SNPs (rs13107662 [*CAMK2D*, *p* = 6.2 × 10^−6^], rs9907521 [*PRKCA,* *p* = 7.1 × 10^−5^], and rs11925452 [*ROBO1, p* = 8.2 × 10^−4^]) were significantly associated with PCa aggressiveness. These alcohol–SNP interactions revealed contrasting effects of excessive alcohol intake on PCa aggressiveness according to the genotypes in the identified SNPs. We identified PCa patients with the rs13107662 (*CAMK2D*) AA genotype, the rs11925452 (*ROBO1*) AA genotype, and the rs9907521 (*PRKCA)* AG genotype were more vulnerable to excessive alcohol intake for developing aggressive PCa. Our findings support that the impact of excessive alcohol intake on PCa aggressiveness was varied by the selected genetic profiles.

## 1. Introduction

Prostate cancer (PCa) is the most common cancer, and the second leading cause of cancer deaths among men in the United States [1] and the sixth cause of cancer-related deaths among men in highly developed regions worldwide [2]. It is commonly accepted that a complex interplay between genetic and environmental factors plays a role in cancer prognosis [3,4]. Both environmental and genetic factors play essential roles in PCa etiology [5,6,7]. Despite many research efforts, the major proportion of the familial risk of PCa remains unknown [8].

Alcohol consumption is considered one of the most important and modifiable environmental risk factors for human cancers, such as liver and colorectal cancers [9]. Alcohol intake is also one of the potentially modifiable factors associated with PCa aggressiveness and prognosis. Among adult Americans, 55.3% consumed alcohol, while 26.5% and 6.6% reported binge and heavy drinking, respectively, in the past month based on the 2018 National Survey on Drug Use and Health [10]. PCa patients have similar excessive alcohol intake compared with non-cancer individuals [11]. The heavy alcohol intake rate in 2019 is similar for PCa patients (4.2%) and non-cancer individuals (5.2%), and the frequent binge alcohol drinking is 4.9% and 4.2% for PCa patients and non-cancer individuals, respectively. Frequent binge drinking was defined as ≥4 binge drinking days (≥5 for males or ≥4 for females of alcohol drinks per day) per month in the past year [11]. Demoury et al. conducted a large population-based case-control study and found high beer intake was associated with PCa aggressiveness [12]. Additionally, a meta-analysis study revealed alcohol consumption to be associated with an increased risk of PCa aggressiveness [13]. However, associations between alcohol use and PCa aggressiveness remain inconclusive [14,15]. 

Genetic variation has been recognized as a risk factor for PCa aggressiveness. Approximately 40 single nucleotide polymorphisms (SNPs), inherited genetic variants, were suggested to be associated with PCa progression in genome-wide association studies (GWASs) [16]. Several SNPs in alcohol-metabolizing genes (*ALDH1A2* and *ALDH1B1*) were associated with PCa specific mortality [17]. Studies on gene–environment interactions can help discover new environmental factors impacting diseases and identify novel genes and high-risk individuals (23–25). Genetic variants may influence alcohol’s impact on PCa aggressiveness. Several pathogenetic mechanisms for alcohol-induced carcinogenesis have been reported [18]. However, the effects of crosstalk between alcohol use and inherited genetic variants on PCa aggressiveness remain understudied.

Accumulating evidence suggests that interplay among angiogenesis, mitochondria, miRNA, and androgen metabolism-related pathways may play a critical role in PCa aggressiveness [19,20,21]. For crosstalk between angiogenesis and androgen, expression of androgen, epigenetic factors, and oxygen level in tumor micro-environment regulates angiogenesis, which leads to metastatic PCa [22]. Recurrent patients who had therapies targeting both angiogenesis and androgen resulted in increasing survival [23]. In addition, androgens influence angiogenesis in androgen-sensitive prostate tumors [24]. These findings suggested the relationship between androgen and angiogenesis and how they interact in aggressive PCa patients. Genes involved in the androgen metabolism pathway also lead to oncogenic metabolic phenotypes, such as mitochondrial respiration and cell proliferation in PCa cells. In addition, androgen repression in PCa cells decreases mitochondrial activity [25]. We and others have reported that miRNAs (such as miR-221, miR-222, and miR-155) are involved in the regulation of various aspects of angiogenesis [20] and progression of PCa [26]. Thus, this study aims to evaluate alcohol intake and explore the alcohol–SNP interactions of the SNPs in the four PCa-related pathways (angiogenesis, mitochondria, miRNA, and androgen metabolism-related pathways) associated with PCa aggressiveness.

## 2. Materials and Methods

### 2.1. Study Population

This study included 3306 PCa patients with European ancestry from the Collaborative Oncological Gene–Environment Study (COGS) in the Prostate Cancer Association Group to investigate Cancer Associated Alterations in the Genome (PRACTICAL) Consortium. In this study, PCa aggressiveness is defined as Gleason score ≥8, Prostate Specific Antigen (PSA) >100, distant disease stage at diagnosis, or death from PCa. Ethnic groups were determined based on ~37,000 uncorrelated markers that passed quality control, including ~1000 selected as ancestry informative markers. European ancestry was defined as ≥85% European component by multidimensional scaling using the three HapMap 2 populations [27]. We randomly assigned half of the participants to the discovery and validation set with a sample size of 1636 and 1670, respectively. The combined set is the sum of the discovery and validation set. There were seven study sites, and we combined the small sites based on geographic region into five integrated study sites (Table S1) for modeling. Details of the PRACTICAL Consortium study have been previously reported [27]. This project was approved by the Louisiana State University Health Sciences Center Institutional Review Boards (IRB #9338).

### 2.2. SNP Selection

This study included 7501 SNPs in the four PCa-related pathways (angiogenesis, mitochondria, miRNA, and androgen metabolism) from the PRACTICAL COGS project. The SNPs with a minor allele frequency (MAF) <0.05 and call rates <95% were excluded. For each SNP, we let a lowercase letter ‘a’ to denote the minor (low frequency) allele and an uppercase ‘A’ to denote the major allele. Each SNP potentially has three genotype categories: homozygous major type (‘AA’), heterozygous type (‘Aa’), and homozygous minor type (‘aa’). The additive mode treated an SNP as a continuous variable by counting the number of minor alleles (AA = 0, Aa = 1, and aa = 2). The comparison of Aa/aa versus AA was performed for the dominant mode, and the comparison of aa versus AA/Aa was made for the recessive mode.

### 2.3. Alcohol Intake Behaviors

We evaluated three excessive alcohol intake behaviors: heavy alcohol intake, heavy beer intake, and high ethanol intake. Heavy alcohol intake was defined as ≥2 times alcohol consumption per day [28]. Heavy beer intake was defined as ≥1 time beer consumption per day [29]. High ethanol intake was defined as intake ≥30 g of alcohol (similar to the recommended limit of two servings) per day [30].

### 2.4. Statistical Analyses

The PCa patients’ alcohol intake and smoking behaviors by PCa aggressiveness (yes/no) and study sets (discovery, validation, and combined set) were summarized using descriptive statistics. We tested the 7501 candidate SNPs in the four target pathways. The selected SNPs, alcohol intake, and smoking behaviors associated with PCa aggressiveness were tested using the Chi-square test or Fisher’s exact test. In order to control for population substructure, principal component analysis was performed, with the first six principal components of population stratification, which was applied as suggested by the PRACTICAL study [27]. Logistic regressions were applied to evaluate alcohol intake associated with PCa aggressiveness adjusting for the study site, the first six principal components, and smoking status. For testing an alcohol–SNP interaction on PCa aggressiveness, the full interaction logistic model with one alcohol intake term, one SNP, and their interaction term was applied. For each SNP, three different inheritance modes (additive, dominant, and recessive) based on the minor allele were tested, and the results with the lowest *p*-value were selected.

**Figure 1:**
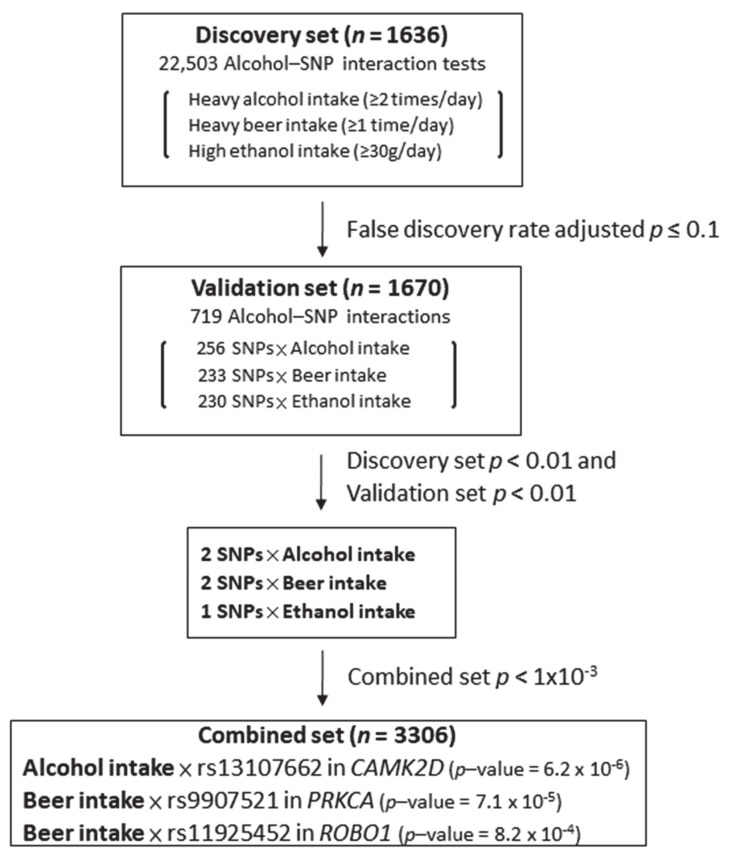
Process of identifying alcohol–single nucleotide polymorphism (SNP) interactions associated with prostate cancer aggressiveness. Note: *p*-values were based on the logistic model adjusting for study site, the first six principal components, and smoking status.

We applied the multi-stage approach (discovery and validation stage, Figure 1) to identify and verify significant alcohol–SNP interactions. In the discovery stage, we used the false discovery rate method to adjust for multiple comparisons [31]. First, alcohol–SNP interaction effect analyses associated with PCa aggressiveness were performed in the discovery set. Interactions with a false discovery rate adjusted *p*-value ≤ 0.10 in the discovery set were further tested in the validation set. Some interactions may have an opposite effect in the discovery and validation sets, so we retested the promising interactions (with *p* < 0.01 in both sets) in the combined set. We defined the significant and verified alcohol–SNP interactions with *p* < 0.01 in both discovery and validation sets and *p* < 0.001 in the combined set. For significant alcohol–SNP interactions, we performed subgroup analyses for evaluating associations between alcohol intake and PCa aggressiveness within the selected genotypes. We also reported the top alcohol–SNP interactions by expanding the *p*-value criteria in the validation set (*p* < 0.05). Smoking is a well-known environmental factor interacting with alcohol intake, so all models for testing alcohol effects, SNP individual effects, and alcohol–SNP interactions were adjusted for the study site, the first six principal components, and smoking. Data analyses were performed using SAS and SNPassoc R package.

## 3. Results

The prevalence of PCa aggressiveness was 17.7%, 17.5%, and 17.9% in the combined, discovery, and validation sets, respectively. In the combined set (Table 1), 29.6%, 20.0%, and 28.2% of PCa patients reported heavy alcohol intake, heavy beer intake, and high ethanol intake, respectively, and 10% of them were current smokers. As shown in Table 1 and Table S2, there were no significant associations between these alcohol intake factors and PCa aggressiveness in the combined, discovery, and validation sets. The prevalence of PCa aggressiveness was the same (20.5%) in the combined set for those with and without heavy alcohol intake. The prevalence of PCa aggressiveness was similar (17.4–20.2%) regardless of beer and ethanol intake status. In contrast, smoking status was significantly associated with PCa aggressiveness in the validation set (*p* = 0.04), but not in the discovery and combined set. In the validation set, current smokers had a higher prevalence of PCa aggressiveness than non-smokers and former smokers (24.7% vs. 16.1% and 17.8%). Similarly, heavy alcohol intake, heavy beer intake, and high ethanol intake showed no significant associations with PCa aggressiveness in the discovery, validation, and combined set after adjusting for study site, the first six principal components, and smoking status (Table S2).

To test whether SNPs in the four pathways influenced the associations between excessive alcohol intake impact and PCa aggressiveness, we evaluated a total of 7501 alcohol–SNP interaction tests for each of the alcohol outcomes in the discovery set. As shown in Figure 1, there were 719 alcohol–SNP interactions (256 for heavy alcohol intake, 233 for heavy beer intake, and 230 for high ethanol intake) with a false discovery rate adjusted *p*-value ≤ 0.10 in the discovery set. These interactions were retested in the validation set. There were five alcohol–SNP interactions with a *p* < 0.01 in both the discovery and validation set (two for heavy alcohol intake, two for heavy beer intake, and one for high ethanol intake). Two of them had an opposite effect in the discovery and validation set, so these two interactions became insignificant in the combined set. Although none of the alcohol–SNP interactions reached the Bonferroni significance level (*p* < 2.2 × 10^−6^ = 0.05/22503), the top three alcohol–SNP interactions were validated and had *p* < 0.001 in the combined set: alcohol–rs13107662 (*p* = 6.2 × 10^−6^), beer–rs9907521 (*p* = 7.1 × 10^−5^), and beer–rs11925452 (*p* = 8.2 × 10^−4^).

As shown in Table 2, the interaction of rs13107662 (*CAMK2D*) and heavy alcohol intake was significantly associated with PCa aggressiveness (*p* = 6.2 × 10^−6^ in the combined, *p* = 2.1 × 10^−4^ discovery, and *p* = 0.003 validation set). The interaction between heavy beer intake and rs9907521 (*PRKCA*) was significantly associated with PCa aggressiveness (*p* = 7.1 × 10^−5^ in the combined set). In addition, the interaction of heavy beer intake and rs11925452 (*ROBO1*) was significantly associated with PCa aggressiveness (*p* = 8.2 × 10^−4^ in the combined set). For these three alcohol–SNP interactions, all of them selected the additive mode, which means the impact of alcohol intake on PCa aggressiveness varied by the number of the minor allele. For the individual SNP effects for the three SNPs involved in these interactions, only rs13107662 in *CAMK2D* was significantly associated with PCa aggressiveness (*p* = 0.003 in the combined set, Table 3). The PCa patients with the rs13107662 GG genotype had a higher chance of PCa aggressiveness than those with the AA or AG genotypes (30.0%, 19.8%, and 18.5% for GG, AA, and AG genotypes, respectively). The other two SNPs (rs9907521 and rs11925452) did not have significant individual SNP effects associated with PCa aggressiveness (*p* = 0.640 and 0.514 in the combined set, respectively).

**Figure 2:**
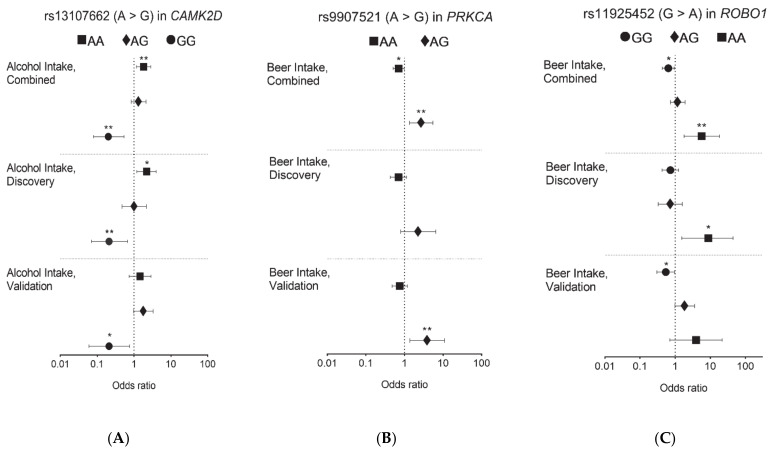
Alcohol intake impact on prostate cancer aggressiveness by the selected genotype profile. (**A**) Heavy alcohol intake impact by genotypes of rs13107662 in *CAMK2D*; (**B**) heavy beer intake impact by genotypes of rs9907521 in *PRKCA*; (**C**) heavy beer intake impact by genotypes of rs11925452 in *ROBO1.* Note: *: *p* < 0.05, **: *p* < 0.01; Logistic models regression adjusted for study site, the six principal components, and smoking status. For a sample size <100 (rs13107662 GG for the discovery and validation set, rs11925452 AA for all sets, and rs9907521GG for all sets), unadjusted results were reported. The order of the genotypes was based on homozygous major, heterozygous, and homozygous minor types.

**Figure 3:**
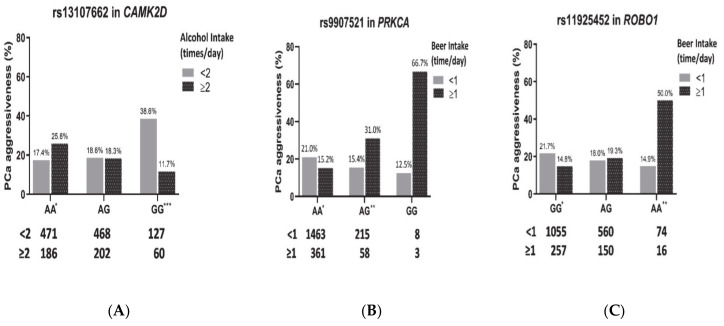
Prevalence of prostate cancer aggressiveness by the selected SNPs and alcohol intake behaviors. (**A**) Interaction of heavy alcohol intake and rs13107662 in *CAMK2D*; (**B**) interaction of heavy beer intake and rs9907521 in *PRKCA*; (**C**) interaction of heavy beer intake rs11925452 in *ROBO1*. Note: *: *p* < 0.05, **: *p* < 0.01, ***: *p* < 0.001.

For these three significant alcohol–SNP interactions, we performed subgroup analyses to evaluate the effects of alcohol intake on PCa aggressiveness within each genotype. For rs13107662 in *CAMK2D* (A > G representing for major “A” allele and minor “G” allele), heavy alcohol intake associated with PCa aggressiveness was significant for PCa patients with the AA and GG genotypes (Figure 2A). For 43.4% PCa patients with the rs13107662 AA genotype (Table 3), those with heavy alcohol intake had a higher chance of PCa aggressiveness (25.8%) compared with those without heavy alcohol intake (17.4%, *p* = 0.015). For PCa patients with the rs13107662 GG genotype, PCa aggressiveness prevalence was 38.6% and 11.7% for low and heavy alcohol intake, respectively (*p* = 1.8 × 10^−4^). Adjusting for the potential confounding factors (study site, the first six principal components, and smoking status), these effects of heavy alcohol intake remained significant. As shown in Figure 3 and Table S3, heavy alcohol intake had a significant risk effect on PCa aggressiveness (odds ratio (OR) = 1.83, *p* = 0.008 in the combined set) for the rs13107662 AA genotype group, but a significantly protective effect on PCa aggressiveness (OR = 0.2, *p* = 0.002) for those with the rs13107662 GG genotype group. Similar trends of this alcohol–SNP interaction can be observed for both the discovery and validation set.

For rs9907521 in *PRKCA* (Figure 2B), heavy beer intake had a significant risk effect on PCa aggressiveness for those with the AG genotype, but a significantly protective effect for those with the AA genotype. For the rs9907521 common AA genotype, PCa aggressiveness prevalence was 21.0% and 15.2% for those with low and heavy beer intake, respectively (*p* = 0.014). For the PCa patients with the rs9907521 AG genotype, PCa aggressiveness prevalence was significantly higher for those with heavy beer intake than those without (31.0% vs. 15.4%, *p* = 0.007). Beer intake effects remained significant after adjusting for the selected factors. For rs9907521 in *PRKCA* (Figure 3 and Table S4), patients with heavy beer intake were more likely to have PCa aggressiveness in the AG genotype group (OR = 2.71, *p* = 0.006 in the combined set), but less likely to have PCa aggressiveness in the AA genotype group (OR = 0.71, *p* = 0.036 in the combined set).

For rs11925452 in *ROBO1* (G > A, Figure 2C), heavy beer intake was significantly associated with PCa aggressiveness for those with the GG and AA genotypes. For 62.1% of PCa patients with the rs11925452 GG genotype (Table 3), heavy beer intake had a protective effect on PCa aggressiveness. In PCa patients with the rs11925452 AA genotype, those with heavy beer intake had a higher chance of PCa aggressiveness than those with low beer intake (50.0% vs. 14.9%, *p* = 0.004). The opposite effect was observed in the GG genotype. The prevalence of PCa aggressiveness was 14.8% and 21.7% for those with heavy and low beer intake, respectively (*p* = 0.014), for those with the rs11925452 GG genotype. Similarly, these beer effects were validated and remained significant after adjusting other factors (Figure 3). Heavy beer intake had a significant risk effect on PCa aggressiveness for PCa patients with the rs11925452 AA genotype (OR = 5.73, *p* = 0.004 in the combined set), but had a significant protective effect for those with the GG genotype (OR = 0.64, *p* = 0.023 in the combined set, Table S5) after adjusting for study site, the first six principal components, and smoking status. In addition to these top three pairs, there were five extra alcohol-SNP pairs by expanding the *p*-value criterion as *p* < 0.05 in the validation set (Table S6). These five alcohol–SNP interactions were beer–rs5745616 (*HGF, p =* 2.0 × 10^−4^), ethanol–rs2050143 (*PDGFB*, *p* = 2.0 × 10^−4^), beer–rs4744514 (*SYK*, *p* = 3.0 × 10^−4^), alcohol–rs11226159 (*PDGFD*, *p* = 5.0 × 10^−4^), and alcohol–rs10933175 (*COL4A3*, *p* = 8.0 × 10^−4^).

## 4. Discussion

This study examined the alcohol–SNP interactions in the four pathways (angiogenesis, mitochondria, miRNA, and androgen metabolism) associated with PCa aggressiveness. Our results showed that excessive alcohol intake factors (≥2 times alcohol intake per day, ≥1 time beer intake per day, and ≥30 g of alcohol per day) were not associated with PCa aggressiveness. However, the interactions of excessive alcohol intake and three SNPs (rs13107662 in *CAMK2D*, rs9907521 in *PRKCA,* and rs11925452 in *ROBO1*) had a significant effect on PCa aggressiveness. Among these three SNPs, only the rs13107662 had a significant individual effect associated with aggressive PCa (Table 3). These significant alcohol–SNP interactions suggested that excessive alcohol intake significantly impacts PCa aggressiveness on the specific genetic sub-groups in *CAMK2D*, *PRKCA*, and *ROBO1*. For all three identified SNPs, excessive alcohol intake had a significant risk effect for one genotype, but had a protective impact on another genotype within the same SNP. Using rs13107662 in *CAMK2D* as an example, high alcohol intake (≥2 times alcohol intake per day) was not significantly associated with PCa aggressiveness, but this alcohol effect varied by rs13107662 genotype status. High alcohol intake had a risk effect on PCa aggressiveness for PCa patients with the rs13107662 AA genotype, but had a protective effect for those with the GG genotype.

The literature supports the biological functions of our three identified alcohol–SNP interactions. Studies reported that *CAMK2D*, *PRKCA,* and *ROBO1* were associated with both alcohol intake and PCa. *CAMK2D* is part of a larger family of CAMKII, a key component in the common five pathways involved in alcohol, cocaine, opioids, and nicotine addiction [32]. CAMKII has been associated with increased frequency of alcohol consumption and drinking patterns [33]. Gene expression of *CAMK2D* is differentially expressed in cocaine-addicted individuals and controls [34]. In addition, one *CAMK2D* genetic variant (rs3815072) is significantly associated with pathological gambling [35]. *CAMK2D* is also overexpressed with ethanol exposure in rats [36]. In addition, gene expression of CAMK2D, enriched in the cell cycle and the calcium signaling pathway, is associated with PCa metastasis [37]. It has been shown that an elevated calcium concentration may facilitate the metastasis of PCa by bone remodeling and the Akt signaling pathway [38]. In addition, CAMK2D appeared to be regulated by miRNA-30, known as a tumor suppressor miRNA. miRNA-30 inhibits cell migration and invasion and is generally under-expressed in PCa tissues [39]. Although there is no report on a role of *CAMK2D* SNP in PCa, two SNPs were investigated as a biomarker for other cancers. rs13107662 was found to be associated with ovarian cancer risk [40], and rs10023113 was found as a prognostic marker for survival of lung cancer patients [41]. 

Our results show that PCa patients with heavy beer intake are more likely to have PCa aggressiveness in the rs9907521 AG genotype in *PRKCA*. Although the risk effect of heavy beer intake was not significant for those with the rs9907521 GG genotype because of the small sample size (*n* = 13), the difference in PCa aggressiveness prevalence for those with and without heavy beer intake (66.7% vs. 12.5%) is the largest among all genotypes for these three SNPs in Figure 2. *PRKCA* (protein kinase C alpha) plays an essential role in many different cellular processes, such as cell adhesion and cell transformation. *PRKCA* has been associated with alcohol dependence and early onset of alcohol dependence for European Americans and African Americans in the GWAS [42,43]. Four SNPs in *PRKCA* (rs17688881, rs721429, rs7217618, and rs8077110) are associated with alcohol dependence and brain activations [44]. *PRKCA* is also a GWAS identified gene significantly associated with food addiction [45]. Knockout studies in mice suggest that *PRKCA* activity regulates cancer, and *PRKCA* was overexpressed in PCa cells [46]. No study evaluated a role of *PRKCA* SNPs associated with PCa aggressiveness. One study reported that rs11079651 in *PRKCA* was associated with pancreatic cancer risk [47]. A preclinical study showed *PRKCA* expression was significantly decreased in rats exposed to ethanol compared with those exposed to either water or saccharin [48]. In addition, expression of *PRKCA* was changed after ethanol exposure in liver-derived cells [49].

*ROBO1* was shown to be involved with both PCa and alcohol intake. *ROBO1*, a member of the roundabout (ROBO) immunoglobulin superfamily of protein, plays a role in cell migration [50,51] and acts as a tumor suppressor gene [52,53]. *ROBO1* expression was negatively associated with prognosis for PCa risk/metastasis [52,54], breast cancer [53,55], and colorectal cancer [56]. The *ROBO/SLIT* signaling pathway acts as a critical regulator for tumor developmental and pathological processes for several cancers, including PCa [57]. ROBO1 acts like a natural inhibitor of metastasis; therefore, *ROBO1* is considered as a potential cancer therapeutic target [58]. We previously reported several SNP–SNP interactions between *R0BO1* and *MMP16* associated PCa aggressiveness, and the biological function of the *ROBO1–MMP16* interactions was supported by gene expression results [21,59]. *ROBO1* is one gene enriched in the axon guidance pathway, significantly associated with alcoholism and alcohol addiction [60].

We also identified five additional alcohol–SNP interaction pairs associated with PCa aggressiveness by expanding the *p*-value criteria to *p* < 0.05 in the validation set. They are rs5745616 (*HGF*), rs2050143 (*PDGFB*), rs4744514 (*SYK*), rs11226159 (*PDGFD*), and rs10933175 (*COL4A3*). Previous studies indicated that *HGF* significantly increases the proliferation and invasion of prostate tumor cells [61,62,63]. In addition, *HGF* leads to recover from alcohol-induced fatty liver by participation in lipid metabolism [64]. Platelet-derived growth factor F (*PDGF*) signaling plays an important role in cancer risk and progression. The *PDGF* isoforms are composed of four different genes, *PDGFA*, *PDGFB*, *PDGFC,* and *PDGFD* [65]. These genes regulate cell proliferation, transformation, apoptosis, invasion, angiogenesis, and metastasis [66,67,68]. Duan et al. reported that two SNPs of *PDGFB* are associated with pancreatic cancer risk [69]. *SYK* is required for angiogenesis and lymphangiogenesis during embryonic development [70,71]. The role of *SYK* in human cancers is not completely established. *SYK* expression inversely correlates with tumor growth and metastasis in breast cancer [72,73]. In addition, binge alcohol intake could induce *SYK* activation in an animal study [74], and *SYK* was associated with the development of alcoholic liver disease and liver fibrosis [75]. In addition, expression of *COL4A3* was associated with tumor size, higher grade, metastasis, and invasion in several malignancies [76,77,78].

We are aware of the limitations of this study. First, our results were generated from PCa patients with European ancestry, so these findings may not be applied in other racial groups. Second, genetic variations and alcohol interactions may reveal the complexity of aggressiveness of prostate cancer. However, these interactions may not be applied in other cancers. Third, this study did not have detailed beverage types (such as red wine, white wine, and spirits) and may have recall bias of the self-report alcohol intake behaviors. Finally, the biological role of identified SNPs in alcohol metabolism is not known. Therefore, basic research on the function of these genetic variants in alcohol metabolism is warranted. Our study findings can be used to identify high-risk genetic groups of PCa aggressiveness. For men with risky alleles, special attention to alcohol intervention for decreasing alcohol consumption should be considered to prevent PCa progression. Future research with a large sample size and information of various alcohol beverage types will be needed to further validate the identified effects of alcohol–SNP interactions on PCa aggressiveness. In summary, our findings suggest that the impact of alcohol intake on PCa aggressiveness may not be universal in PCa patients owing to the effects of genetic heterogeneity, such as *CAMK2D*, *PRKCA*, and *ROBO1*. These alcohol–SNP interactions may explain the inconclusive results of alcohol’s impact on PCa aggressive when genetic profiles are not considered. These results suggested that excessive alcohol intake significantly impacts PCa aggressiveness in the specific genetic subgroups.

## Figures and Tables

**Table 1:** Alcohol intake and smoking status associated with prostate cancer (PCa) aggressiveness by study sets.

	Combined (*n* = 3306) PCa Aggressiveness (17.7%)			Discovery (*n* = 1636) PCa Aggressiveness (17.5%)		Validation (*n* = 1670) PCa Aggressiveness (17.9%)	
Factors	Total *N* (%)	Yes *N* (%)	No *N* (%)	Yes *N* (%)	No *N* (%)	Yes *N* (%)	No *N* (%)
Heavy alcohol intake (≥2 times/day)							
No Yes	1066 (70.4) 448 (29.6)	218 (20.5) 92 (20.5)	848 (79.5) 356 (79.5)	114 (21.8) 46 (20.8)	410 (78.2) 175 (79.2)	104 (19.2) 46 (20.3)	438 (80.8) 181 (79.7)
Heavy beer intake (≥1 time/day)							
No Yes	1690 (80.0) 423 (20.0)	341 (20.2) 75 (17.7)	1349 (79.8) 348 (82.3)	167 (20.2) 35 (16.6)	659 (79.8) 176 (83.4)	174 (20.1) 40 (18.9)	690 (79.9) 172 (81.1)
High ethanol intake (≥30 g/day)							
No Yes	2375 (71.8) 931 (28.2)	414 (17.4) 171 (18.4)	1961 (82.6) 760 (81.6)	205 (17.3) 81 (17.9)	978 (82.7) 372 (82.1)	209 (17.5) 90 (18.8)	983 (82.5) 388 (81.2)
Smoking status ^1^							
Non-smoker Former smoker Current smoker	1263 (38.6) 1679 (51.4) 327 (10.0)	218 (17.3) 291 (17.3) 71 (21.7)	1045 (82.7) 1388 (82.7) 256 (78.3)	116 (18.4) 138 (16.8) 31 (18.8)	515 (81.6) 683 (83.2) 134 (81.2)	102 (16.1) 153 (17.8) 40 (24.7)	530 (83.9) 705 (82.2) 122 (75.3)

^1^*p* = 0.040 for smoking vs. PCa aggressiveness in the validation set; other factors associated with PCa aggressiveness were not significant (*p*-value > 0.05).

**Table 2:** Three verified alcohol–single nucleotide polymorphism (SNP) interactions associated with prostate cancer aggressiveness.

Alcohol-SNP Interaction	Combined		Discovery		Validation	
	*p*-Value ^1^	Mode ^2^	*p*-Value ^1^	Mode ^2^	*p*-Value ^1^	Mode ^2^
Heavy alcohol Intake–rs13107662 (*CAMK2D*)	6.2 × 10^−6^	Add	2.1 × 10^−4^	Add	0.003	Rec ^4^
Heavy beer Intake–rs9907521 (*PRKCA*)	7.1 × 10^−5^	Add	0.005	Add	0.005	Add
Heavy beer Intake–rs11925452 (*ROBO1*)	8.2 × 10^−4^	Add	0.003	Rec ^3^	0.002	Add

^1^*p*-value of alcohol–SNP interactions were based on logistic regression adjusted for study site, first six principal components, and smoking status. ^2^ Add: additive, Rec: recessive mode; ^3^ *p* = 0.073 for the additive mode; ^4^ *p* = 0.006 for the additive mode.

**Table 3:** SNP individual effects for SNPs involved with alcohol–SNP interactions associated with prostate cancer (PCa) aggressiveness.

KERRYPNX	Combine PCa Aggressiveness (17.7%)			Discovery PCa Aggressiveness (17.5%)		Validation PCa Aggressiveness (17.9%)	
SNP (Gene, Major > Minor Allele, MAF) ^1^	Total *N* (%)	Yes *N* (%)	No *N* (%)	Yes *N* (%)	No *N* (%)	Yes *N* (%)	No *N* (%)
rs13107662 (*CAMK2D, A > G, 34.5%*) ^2^							
AA	657 (43.4)	130 (19.8)	527 (80.2)	74 (21.8)	266 (78.2)	56 (17.7)	261 (82.3)
AG	670 (44.3)	124 (18.5)	546 (81.5)	56 (18.3)	250 (81.7)	68 (18.7)	296 (81.3)
GG	187 (12.4)	56 (30.0)	131 (70.1)	30 (30.3)	69 (69.7)	26 (29.6)	62 (70.5)
		*p* = 0.003		*p* = 0.046		*p* = 0.046	
rs9907521 (*PRKCA, A > G, 7.0%*) ^2^							
AA	1824 (86.5)	362 (19.9)	1462 (80.2)	172 (19.1)	727 (80.9)	190 (20.5)	735 (79.5)
AG	273 (13.0)	51 (18.7)	222 (81.3)	27 (21.1)	101 (78.9)	24 (16.6)	121 (83.5)
GG	11 (0.5)	3 (27.3)	8 (72.7)	3 (33.3)	6 (66.7)	0	2 (100)
		*p* = 0.640		*p* = 0.456		*p* = 0.561	
rs11925452 (*ROBO1, G > A, 21.1%*) ^2^							
GG	1312 (62.1)	267 (20.4)	1045 (79.7)	130 (20.4)	507 (79.6)	137 (20.3)	538 (79.7)
AG	710 (33.6)	130 (18.3)	580 (81.7)	61 (17.1)	295 (82.9)	69 (19.5)	285 (80.5)
AA	90 ( 4.3)	19 (21.1)	71 (78.9)	11 (25.0)	33 (75.0)	9 (17.4)	38 (82.6)
							
AA							
							
AA							
							
AA							
		*p* = 0.514		*p* = 0.274		*p* = 0.889	

^1^ MAF: minor allele frequency. ^2^ *p*-values were based on Fisher’s exact test.

## Data Availability

Restrictions apply to the availability of the data used in this project. Data were obtained from the Prostate Cancer Association Group to Investigate Cancer Associated Alterations in the Genome consortium (PRACTICAL Consortium, http://practical.icr.ac.uk/blog/) and are available with the permission of PRACTICAL Consortium.

## Supplementary Materials

The following are available online at https://www.mdpi.com/2077-0383/10/3/553/s1, Table S1: Study site information; Table S2: Alcohol intake associated with prostate cancer (PCa) aggressiveness by study set; Table S3: Impact of alcohol intake on prostate cancer aggressiveness for the sub-groups with different genotypes of rs13107662 in *CAMK2D*; Table S4: Impact of alcohol intake on prostate cancer aggressiveness for the sub-groups with different genotypes of rs9907521 in *PRKCA*; Table S5: Impact of alcohol intake on prostate cancer aggressiveness for the sub-groups with different genotypes of rs11925452 in *ROBO1*; Table S6. Eight alcohol–SNP interactions associated with prostate cancer aggressiveness based on liberal significance criteria.
