# Supplementary material for: Alcohol Intake and Alcohol–SNP Interactions Associated with Prostate Cancer Aggressiveness
Source: J Clin Med. 2021 Feb 2;10(3):553. doi: 10.3390/jcm10030553 (PMC7867322; doi:10.3390/jcm10030553)
Supplement: Supplementary file 1 [file jcm-10-00553-s001.zip › Supplementary author note to XML.docx]

The PRACTICAL CONSORTIUM (in addition to those named in the author list)

Information of the consortium can be found at http://practical.icr.ac.uk/

Additional members from the consortium are:

Zsofia Kote-Jarai^1^, UKGPCS collaborators^2^, Artitaya Lophatananon^3^, Alison M. Dunning^4^, Maya Ghoussaini^5^, Fredrick R. Schumacher^6,7^, Loic Le Marchand^8^, Xin Sheng^9^, Xin Gao^10^, Bernd Holleczek^11^, Ben Schöttker^10^, Johanna Schleutker^12,13^, Teuvo L. J. Tammela^14^, Csilla Sipeky^12^, Anssi Auvinen^15^, Jyotsna Batra^16, 17^, APCB (Australian Prostate Cancer BioResource)^17, 18^, Suzanne Chambers^19, 20^, Lisa Horvath^21,22^, Leire Moya^17,23^, Gail P. Risbridger^24, 25^, Wayne Tilley^26^, Judith A. Clements^17, 23^, David E. Neal^27, 28, 29^, Jenny L. Donovan^30^, Freddie C. Hamdy^31, 32^, Richard M. Martin^30, 33, 34^, Sune F. Nielsen^35, 36^, Stig E. Bojesen^35, 36^, Peter Iversen^37^, Martin Andreas Røder^37^, Henrik Grönberg^38^, Fredrik Wiklund^38^, Graham G. Giles^39, 40, 41^, Melissa C. Southey^39, 41, 42^, Robert J. MacInnis^39, 40^, Roger L. Milne^39, 40, 41^, Ruth C. Travis^43^, Tim J. Key^43^, William J. Blot^44, 45^, Wei Zheng^44^, Janet L. Stanford^46, 47^, Elaine A. Ostrander^48^, Milan S. Geybels^46^, Børge G. Nordestgaard^35, 36^, Adam S. Kibel^49^, Bettina F. Drake^50^, Markus Aly^38, 51, 52^, Cezary Cybulski^53^, Dominika Wokolorczyk^53^, Jan Lubinski^53^, Kay-Tee Khaw^54^, Christiane Maier^55^, Manuel Luedeke^55^, Thomas Schnoeller^56^, Stephen N. Thibodeau^57^, Shannon K. McDonnell^58^, Daniel J. Schaid^58^, Manuel R. Teixeira^59, 60, 61^, Paula Paulo^61^, Andreia Brandão^59, 61^, Lisa Cannon-Albright^62, 63^, Craig C Teerlink^62, 63^, Radka Kaneva^64^, Chavdar Slavov^65^, Vanio Mitev^64^, Hardev Pandha^66^, Agnieszka Michael^66^

^1^The Institute of Cancer Research, London, SM2 5NG, UK

^2^http://www.icr.ac.uk/our-research/research-divisions/division-of-genetics-and-epidemiology/oncogenetics/research-projects/ukgpcs/ukgpcs-collaborators

^3^Division of Population Health, Health Services Research and Primary Care, School of Health Sciences, Faculty of Biology, Medicine and Health, University of Manchester, Manchester, M13 9PL, UK

^4^Centre for Cancer Genetic Epidemiology, Department of Oncology, University of Cambridge, Strangeways Laboratory, Worts Causeway, Cambridge, CB1 8RN, UK

^5^Open Targets, Wellcome Sanger Institute, Hinxton, Saffron Walden, CB10 1SA, UK

^6^Department of Population and Quantitative Health Sciences, Case Western Reserve University, Cleveland, OH 44106-7219, USA

^7^Seidman Cancer Center, University Hospitals, Cleveland, OH 44106, USA

^8^Epidemiology Program, University of Hawaii Cancer Center, Honolulu, HI 96813, USA

^9^Center for Genetic Epidemiology, Department of Preventive Medicine, Keck School of Medicine, University of Southern California/Norris Comprehensive Cancer Center, Los Angeles, CA 90015, USA

^10^Division of Clinical Epidemiology and Aging Research, German Cancer Research Center (DKFZ), Heidelberg, Germany

^11^Saarland Cancer Registry, 66119 Saarbrücken, Germany

^12^Institute of Biomedicine, Kiinamyllynkatu 10, FI-20014 University of Turku, Finland

^13^Department of Medical Genetics, Genomics, Laboratory Division, Turku University Hospital, PO Box 52, 20521 Turku, Finland

^14^Department of Urology, Tampere University Hospital, Tampere, Finland

^15^Unit of Health Sciences, Faculty of Social Sciences, Tampere University, Tampere, Finland

^16^Australian Prostate Cancer Research Centre-Qld, Institute of Health and Biomedical Innovation and School of Biomedical Sciences, Queensland University of Technology, Brisbane QLD 4059, Australia

^17^Translational Research Institute, Brisbane, Queensland 4102, Australia

^18^The Kinghorn Cancer Centre, Sydney, Australia

^19^University of Technology, Sydney

^20^Cancer Council Queensland, Fortitude Valley, QLD 4006, Australia

^21^Chris O'Brien Lifehouse (COBLH), Camperdown, Sydney, NSW 2010, Australia

^22^Garvan Institute of Medical Research, Sydney NSW 2010, Australia

^23^Australian Prostate Cancer Research Centre-Qld, Institute of Health and Biomedical Innovation and School of Biomedical Sciences, Queensland University of Technology, Brisbane, 4059, Australia

^24^Department of Anatomy and Developmental Biology, Biomedicine Discovery Institute, Monash University, Melbourne, Victoria 3800, Australia

^25^Prostate Cancer Translational Research Program, Cancer Research Division, Peter MacCallum Cancer Centre, Melbourne, VIC 3000, Australia

^26^Dame Roma Mitchell Cancer Research Laboratories, University of Adelaide, Adelaide, South Australia, Australia

^27^Nuffield Department of Surgical Sciences, University of Oxford, Room 6603, Level 6, John Radcliffe Hospital, Headley Way, Headington, Oxford, OX3 9DU, UK

^28^University of Cambridge, Department of Oncology, Box 279, Addenbrooke's Hospital, Hills Road, Cambridge CB2 0QQ, UK

^29^Cancer Research UK, Cambridge Research Institute, Li Ka Shing Centre, Cambridge UK

^30^Population Health Sciences, Bristol Medical School, University of Bristol, BS8 2PS, UK

^31^Nuffield Department of Surgical Sciences, University of Oxford, Oxford, OX1 2JD, UK

^32^Faculty of Medical Science, University of Oxford, John Radcliffe Hospital, Oxford, UK

^33^National Institute for Health Research (NIHR) Biomedical Research Centre, University of Bristol, Bristol, BS8 1TH, UK

^34^Medical Research Council (MRC) Integrative Epidemiology Unit, University of Bristol, Bristol, BS8 2BN, UK

^35^Faculty of Health and Medical Sciences, University of Copenhagen, 2200 Copenhagen, Denmark

^36^Department of Clinical Biochemistry, Herlev and Gentofte Hospital, Copenhagen University Hospital, Herlev, 2200 Copenhagen, Denmark

^37^Copenhagen Prostate Cancer Center, Department of Urology, Rigshospitalet, Copenhagen University Hospital, DK-2730 Herlev, Copenhagen, Denmark

^38^Department of Medical Epidemiology and Biostatistics, Karolinska Institute, Stockholm, Sweden

^39^Cancer Epidemiology Division, Cancer Council Victoria, 615 St Kilda Road, Melbourne, VIC 3004, Australia

^40^Centre for Epidemiology and Biostatistics, Melbourne School of Population and Global Health, The University of Melbourne, Grattan Street, Parkville, VIC 3010, Australia

^41^Precision Medicine, School of Clinical Sciences at Monash Health, Monash University, Clayton, Victoria 3168, Australia

^42^Department of Clinical Pathology, The Melbourne Medical School, The University of Melbourne, Melbourne, Victoria, Australia.

^43^Cancer Epidemiology Unit, Nuffield Department of Population Health, University of Oxford, Oxford, OX3 7LF, UK

^44^Division of Epidemiology, Department of Medicine, Vanderbilt University Medical Center, 2525 West End Avenue, Nashville, TN 37232 USA.

^45^International Epidemiology Institute, Rockville, MD 20850, USA

^46^Division of Public Health Sciences, Fred Hutchinson Cancer Research Center, Seattle, Washington, 98109-1024, USA

^47^Department of Epidemiology, School of Public Health, University of Washington, Seattle, Washington 98195, USA

^48^National Human Genome Research Institute, National Institutes of Health, 50 South Drive, Rm. 5351, Bethesda, MD 20892, USA

^49^Division of Urologic Surgery, Brigham and Womens Hospital, 75 Francis Street, Boston, MA 02115, USA

^50^Washington University School of Medicine, 660 S. Euclid Avenue, Campus Box 8242, St. Louis, MO 63110, USA

^51^Department of Molecular Medicine and Surgery, Karolinska Institutet, and Department of Urology, Karolinska University Hospital, Solna, 171 76 Stockholm, Sweden

^52^Department of Urology, Karolinska University Hospital, Stockholm, Sweden

^53^International Hereditary Cancer Center, Department of Genetics and Pathology, Pomeranian Medical University, Szczecin, Poland

^54^Clinical Gerontology Unit, University of Cambridge, Cambridge, CB2 2QQ, UK

^55^Humangenetik Tuebingen, Paul-Ehrlich-Str 23, D-72076 Tuebingen, Germany

^56^Department of Urology, University Hospital Ulm, Germany

^57^Department of Laboratory Medicine and Pathology, Mayo Clinic, Rochester, MN 55905, USA

^58^Division of Biomedical Statistics & Informatics, Mayo Clinic, Rochester, MN 55905, USA

^59^Department of Genetics, Portuguese Oncology Institute of Porto (IPO-Porto), Porto, Portugal

^60^Biomedical Sciences Institute (ICBAS), University of Porto, Porto, Portugal

^61^Cancer Genetics Group, IPO-Porto Research Center (CI-IPOP), Portuguese Oncology Institute of Porto (IPO-Porto), Porto, Portugal

^62^Division of Epidemiology, Department of Internal Medicine, University of Utah School of Medicine

^63^George E. Wahlen Department of Veterans Affairs Medical Center, Salt Lake City, Utah, USA

^64^Molecular Medicine Center, Department of Medical Chemistry and Biochemistry, Medical University of Sofia, Sofia, 2 Zdrave Str., 1431 Sofia, Bulgaria

^65^Department of Urology and Alexandrovska University Hospital, Medical University of Sofia, 1431 Sofia, Bulgaria

^66^The University of Surrey, Guildford, Surrey, GU2 7XH, UK

**Funding for the CRUK study and PRACTICAL consortium:**

This work was supported by the Canadian Institutes of Health Research, European Commission's Seventh Framework Programme grant agreement n° 223175 (HEALTH-F2-2009-223175), Cancer Research UK Grants C5047/A7357, C1287/A10118, C1287/A16563, C5047/A3354, C5047/A10692, C16913/A6135, and The National Institute of Health (NIH) Cancer Post-Cancer GWAS initiative grant: No. 1 U19 CA 148537-01 (the GAME-ON initiative).

**COGS acknowledgement:**

This study would not have been possible without the contributions of the following: Per Hall (COGS); Douglas F. Easton, Paul Pharoah, Kyriaki Michailidou, Manjeet K. Bolla, Qin Wang (BCAC), Andrew Berchuck (OCAC), Rosalind A. Eeles, Douglas F. Easton, Ali Amin Al Olama, Zsofia Kote-Jarai, Sara Benlloch (PRACTICAL), Georgia Chenevix-Trench, Antonis Antoniou, Lesley McGuffog, Fergus Couch and Ken Offit (CIMBA), Joe Dennis, Alison M. Dunning, Andrew Lee, and Ed Dicks, Craig Luccarini and the staff of the Centre for Genetic Epidemiology Laboratory, Javier Benitez, Anna Gonzalez-Neira and the staff of the CNIO genotyping unit, Jacques Simard and Daniel C. Tessier, Francois Bacot, Daniel Vincent, Sylvie LaBoissière and Frederic Robidoux and the staff of the McGill University and Génome Québec Innovation Centre, Stig E. Bojesen, Sune F. Nielsen, Borge G. Nordestgaard, and the staff of the Copenhagen DNA laboratory, and Julie M. Cunningham, Sharon A. Windebank, Christopher A. Hilker, Jeffrey Meyer and the staff of Mayo Clinic Genotyping Core Facility

Funding for the iCOGS infrastructure came from: the European Community's Seventh Framework Programme under grant agreement n° 223175 (HEALTH-F2-2009-223175) (COGS), Cancer Research UK (C1287/A10118, C1287/A 10710, C12292/A11174, C1281/A12014, C5047/A8384, C5047/A15007, C5047/A10692, C8197/A16565), the National Institutes of Health (CA128978) and Post-Cancer GWAS initiative (1U19 CA148537, 1U19 CA148065 and 1U19 CA148112 - the GAME-ON initiative), the Department of Defence (W81XWH-10-1-0341), the Canadian Institutes of Health Research (CIHR) for the CIHR Team in Familial Risks of Breast Cancer, Komen Foundation for the Cure, the Breast Cancer Research Foundation, and the Ovarian Cancer Research Fund.

**Additional funding and acknowledgments from studies in PRACTICAL that provided data for this analysis:**

ESTHER

(Epidemiological investigations of the chances of preventing, recognizing early and optimally treating chronic diseases in an elderly population)

The ESTHER study was supported by a grant from the Baden Württemberg Ministry of Science, Research and Arts.

The ESTHER group would like to thank Hartwig Ziegler, Sonja Wolf, Volker Hermann, Heiko Müller, Karina Dieffenbach, Katja Butterbach for valuable contributions to the study.

MEC

(Multiethnic Cohort Study)

The MEC was supported by NIH grants CA063464, CA054281, CA098758, and CA164973.

MOFFITT

(The Moffitt Group)

The Moffitt group was supported by the US National Cancer Institute (R01CA128813, PI: J.Y. Park).

SEARCH

(Study of Epidemiology and Risk factors in Cancer Heredity)

SEARCH is funded by a programme grant from Cancer Research UK [C490/A10124] and supported by the UK National Institute for Health Research Biomedical Research Centre at the University of Cambridge. The University of Cambridge has received salary support in respect of PP from the NHS in the East of England through the Clinical Academic Reserve.

UKGPCS

(U.K. Genetic Prostate Cancer Study)

UKGPCS would also like to thank the following for funding support: The Institute of Cancer Research and The Everyman Campaign, The Prostate Cancer Research Foundation, Prostate Research Campaign UK (now Prostate Action), The Orchid Cancer Appeal, The National Cancer Research Network UK, The National Cancer Research Institute (NCRI) UK. We are grateful for support of NIHR funding to the NIHR Biomedical Research Centre at The Institute of Cancer Research and The Royal Marsden NHS Foundation Trust. UKGPCS should also like to acknowledge the NCRN nurses, data managers and Consultants for their work in the UKGPCS.

UKGPCS would like to thank all urologists and other persons involved in the planning, coordination, and data collection of the study. KM and AL were in part supported from the NIHR Manchester Biomedical Research Centre
