# Supplementary material for: Alcohol Intake and Alcohol–SNP Interactions Associated with Prostate Cancer Aggressiveness
Source: J Clin Med. 2021 Feb 2;10(3):553. doi: 10.3390/jcm10030553 (PMC7867322; doi:10.3390/jcm10030553)
Supplement: Supplementary file 1 [file jcm-10-00553-s001.zip › Supplementary Materials to XML.docx]

**Supplementary Materials**

**Table 1.** Study site information.

| **Study site** | **Total**  ***N*** |
| --- | --- |
| Moffitt Cancer Center, Tampa, USA | 249 |
| University of Southern California, Los Angeles, USA | 517 |
| German Cancer Research Center, Heidelberg, Germany | 278 |
| University College London, London, UK | 1068 |
| The Institute of Cancer Research, London, UK & University of Manchester, Manchester, UK | 1194 |
| **Total** | **3306** |

**Table 2.** Alcohol intake associated with prostate cancer (PCa) aggressiveness by study set.

| \|  \| Combined set \| Discover set \| Validation set \| \| --- \| --- \| --- \| --- \| \| Alcohol intake \| Adjusted OR (95% CI)^1^ \| Adjusted OR (95% CI)^1^ \| Adjusted OR (95% CI)^1^ \| \| Heavy alcohol intake (≥ 2 times/day) \| \|  \|  \| \| No \| 1 \| 1 \| 1 \| \| Yes \| 1.12 (0.83, 1.51) \| 1.01 (0.66, 1.55) \| 1.18 (0.76, 1.82) \| \| Heavy beer intake (≥ 1 time/day) \| \|  \|  \| \| No \| 1 \| 1 \| 1 \| \| Yes \| 0.88 (0.66, 1.18) \| 0.87 (0.57, 1.32) \| 0.94 (0.63, 1.42) \| \| High ethanol intake (≥30g/day) \| \|  \|  \| \| No \| 1 \| 1 \| 1 \| \| Yes \| 1.01 (0.82, 1.24) \| 0.94 (0.70, 1.27) \| 1.08 (0.81, 1.45) \| |
| --- | --- | --- | --- | --- | --- | --- | --- | --- | --- | --- | --- | --- | --- | --- | --- | --- | --- | --- | --- | --- | --- | --- | --- | --- | --- | --- | --- | --- | --- | --- | --- | --- | --- | --- | --- | --- | --- | --- | --- | --- | --- | --- | --- | --- |

^1^OR: odds ratio of prostate cancer aggressiveness, CI: confidence interval. One alcohol factor was included in the logistic model adjusting for study site, the first 6 principal components, and smoking status. The alcohol intake factors had *p* > 0.05 for all models.

**Table 3.** Impact of alcohol intake on prostate cancer aggressiveness for the sub-groups with different genotypes of rs13107662 in *CAMK2D.*

| rs13107662 in *CAMK2D* | | | *n* | OR of PCa aggressiveness (95% CI)^1^ | *p*-value^2^ |
| --- | --- | --- | --- | --- | --- |
| Heavy alcohol intake (≥ 2 times/day), Combined | | | |  |  |
|  | AA | 657 | | **1.83 (1.17, 2.85)** | **0.008** |
|  | AG | 670 | | 1.33 (0.83, 2.13) | 0.227 |
|  | GG | 187 | | **0.20 (0.08, 0.54)** | **0.002** |
| Heavy alcohol intake (≥ 2 times/day), Discovery | | | |  |  |
|  | AA | 340 | | **2.19 (1.20, 4.00)** | **0.010** |
|  | AG | 306 | | 1.01 (0.47, 2.19) | 0.977 |
|  | GG | 99 | | **0.21 (0.07, 0.67)** | **0.009** |
| Heavy alcohol intake (≥ 2 times/day), Validation | | | |  |  |
|  | AA | 317 | | 1.45 (0.73, 2.89) | 0.288 |
|  | AG | 364 | | 1.80 (0.97, 3.33) | 0.063 |
|  | GG | 88 | | **0.21 (0.06, 0.76)** | **0.018** |
| Heavy beer Intake (≥ 1 time/day), Combined | | | |  |  |
|  | AA | 937 | | 1.15 (0.76, 1.75) | 0.500 |
|  | AG | 913 | | 0.86 (0.54, 1.39) | 0.547 |
|  | GG | 263 | | 0.47 (0.21, 1.05) | 0.066 |
| High ethanol intake (≥30g/day), Combined | | | |  |  |
|  | AA | 1466 | | 1.07 (0.79, 1.47) | 0.659 |
|  | AG | 1431 | | 1.23 (0.90, 1.69) | 0.198 |
|  | GG | 409 | | 0.61 (0.35, 1.07) | 0.082 |

^1^ OR (odds ratio), CI (confidence interval) based on logistic regression adjusting for study site, the first six principal components, and smoking status. For a sample size less than 100, unadjusted results were reported;^2^ *p* < 0.05 were bold.

**Table 4.** Impact of alcohol intake on prostate cancer aggressiveness for the sub-groups with different genotypes of rs9907521 in *PRKCA.*

| rs9907521 in *PRKCA* | | *n* | OR of PCa aggressiveness (95% CI)^1^ | *p*-value^2^ |
| --- | --- | --- | --- | --- |
| Heavy beer intake (≥ 1 time/day), Combined | | | |  |
|  | AA | 1824 | **0.71 (0.51, 0.98)** | **0.036** |
|  | AG | 273 | **2.71 (1.34,5.50)** | **0.006** |
|  | GG | 11 | NA | NA |
| Heavy beer intake (≥ 1 time/day), Discovery | | | |  |
|  | AA | 899 | 0.70 (0.43, 1.13) | 0.138 |
|  | AG | 128 | 2.28 (0.80, 6.49) | 0.123 |
|  | GG | 9 | NA | NA |
| Heavy beer intake (≥ 1 time/day), Validation | | | |  |
|  | AA | 925 | 0.76 (0.48, 1.19) | 0.225 |
|  | AG | 145 | **3.89 (1.39, 10.84)** | **0.010** |
|  | GG | 2 | NA | NA |
| Heavy alcohol intake (≥ 2 times/day), Combined | | | |  |
|  | AA | 1305 | 1.02 (0.74, 1.42) | 0.885 |
|  | AG | 197 | 1.98 (0.89, 4.40) | 0.093 |
|  | GG | 8 | NA | NA |
| High ethanol intake (≥30g/day), Combined | | | |  |
|  | AA | 2859 | 0.95 (0.76, 1.18) | 0.632 |
|  | AG | 429 | 1.35 (0.77, 2.35) | 0.295 |
|  | GG | 13 | NA | NA |

^1^ OR (odds ratio), CI (confidence interval) based on logistic regression adjusting for study site, the first six principal components, and smoking status. For a sample size less than 100, unadjusted results were reported;^2^ *p* < 0.05 were bold.

**Table 5.** Impact of alcohol intake on prostate cancer aggressiveness for the sub-groups with different genotypes of rs11925452 in *ROBO1.*

| rs11925452 in *ROBO1* | | *n* | OR of PCa aggressiveness (95% CI)^1^ | *p*-value^2^ |
| --- | --- | --- | --- | --- |
| Heavy beer intake (≥ 1 time/day), Combined | | |  |  |
|  | AA | 90 | **5.73 (1.78, 18.47)** | **0.004** |
|  | AG | 710 | 1.18 (0.73, 1.91) | 0.492 |
|  | GG | 1312 | **0.64 (0.43, 0.94)** | **0.023** |
| Heavy beer intake (≥ 1 time/day), Discovery | | |  |  |
|  | AA | 44 | **8.83 (1.55, 44.64)** | **0.013** |
|  | AG | 356 | 0.73 (0.33, 1.60) | 0.427 |
|  | GG | 637 | 0.73 (0.42, 1.26) | 0.262 |
| Heavy beer intake (≥ 1 time/day), Validation | | |  |  |
|  | AA | 46 | 3.96 (0.71, 21.96) | 0.115 |
|  | AG | 354 | 1.87 (0.99, 3.57) | 0.056 |
|  | GG | 675 | **0.54 (0.30, 0.96)** | **0.034** |
| Heavy alcohol intake (≥ 2 times/day), Combined | | |  |  |
|  | AA | 60 | 2.04 (0.62, 6.66) | 0.239 |
|  | AG | 498 | 1.00 (0.58, 1.74) | 0.999 |
|  | GG | 956 | 1.15 (0.79, 1.67) | 0.448 |
| High ethanol intake (≥30g/day), Combined | | |  |  |
|  | AA | 141 | 2.13 (0.74, 6.18) | 0.162 |
|  | AG | 1140 | 1.18 (0.83, 1.68) | 0.367 |
|  | GG | 2024 | 0.92 (0.71, 1.19) | 0.530 |

^1^ OR (odds ratio), CI (confidence interval) based on logistic regression adjusting for study site, the first six principal components, and smoking status. For a sample size less than 100, unadjusted results were reported;^2^ *p* < 0.05 were bold.

**Table 6.** Eight alcohol-SNP interactions associated with prostate cancer aggressiveness based on liberal significance criteria^1.^

| **Alcohol-SNP interaction** | **Combined** | |  | **Discovery** | |  | **Validation** | |
| --- | --- | --- | --- | --- | --- | --- | --- | --- |
|  | ***p*-value^2^** | **mode^3^** |  | ***p*-value^2^** | **mode^3^** |  | ***p*-value^2^** | **mode^3^** |
| Heavy alcohol Intake - rs13107662 (*CAMK2D*) | 6.2x10^−6^ | Add |  | 2.1x10^−4^ | Add |  | 0.003 | Rec |
| Heavy beer Intake - rs9907521 (*PRKCA*) | 7.1x10^−5^ | Add |  | 0.005 | Add |  | 0.005 | Add |
| Heavy beer Intake - rs11925452 (*ROBO1*) | 8.2x10^−4^ | Add |  | 0.003 | Rec |  | 0.002 | Add |
| Heavy beer Intake -rs5745616 (*HGF*) | 2.0x10^−4^ | Dom |  | 0.005 | Dom |  | 0.021 | Dom |
| High ethanol intake -rs2050143 (*PDGFB*) | 2.0x10^−4^ | Rec |  | 0.009 | Rec |  | 0.011 | Add |
| Heavy beer Intake -rs4744514 (*SYK*) | 3.0x10^−4^ | Rec |  | 0.008 | Rec |  | 0.015 | Rec |
| Heavy alcohol intake -rs11226159 (*PDGFD*) | 5.0x10^−4^ | Rec |  | 0.007 | Rec |  | 0.019 | Rec |
| Heavy alcohol intake -rs10933175 (*COL4A3*) | 8.0x10^−4^ | Dom |  | 0.007 | Dom |  | 0.043 | Add |

^1^*p*_discovery < 0.01, *p*_validation < 0.05 and *p*_combined < 1x10^-3^. ^2^*p*-value of alcohol-SNP interactions were based on logistic regression adjusted for study site, first six principal components, and smoking status. ^3^Add: additive, Dom: dominant, and Rec: recessive mode
